# Effects of acute spinalization on neurons of postural networks

**DOI:** 10.1038/srep27372

**Published:** 2016-06-15

**Authors:** Pavel V. Zelenin, Vladimir F. Lyalka, Li-Ju Hsu, Grigori N. Orlovsky, Tatiana G. Deliagina

**Affiliations:** 1 Department of Neuroscience, Karolinska Institute, SE-17177, Stockholm, Sweden

## Abstract

Postural limb reflexes (PLRs) represent a substantial component of postural corrections. Spinalization results in loss of postural functions, including disappearance of PLRs. The aim of the present study was to characterize the effects of acute spinalization on two populations of spinal neurons (F and E) mediating PLRs, which we characterized previously. For this purpose, in decerebrate rabbits spinalized at T12, responses of interneurons from L5 to stimulation causing PLRs before spinalization, were recorded. The results were compared to control data obtained in our previous study. We found that spinalization affected the distribution of F- and E-neurons across the spinal grey matter, caused a significant decrease in their activity, as well as disturbances in processing of posture-related sensory inputs. A two-fold decrease in the proportion of F-neurons in the intermediate grey matter was observed. Location of populations of F- and E-neurons exhibiting significant decrease in their activity was determined. A dramatic decrease of the efficacy of sensory input from the ipsilateral limb to F-neurons, and from the contralateral limb to E-neurons was found. These changes in operation of postural networks underlie the loss of postural control after spinalization, and represent a starting point for the development of spasticity.

In quadrupeds, deviations of the trunk from the dorsal-side-up orientation cause postural corrections, which are generated mainly on the basis of signals from limb mechanoreceptors^1^,^2^,^3^. Earlier, we studied postural limb reflexes (PLRs) in decerebrate rabbits. We have found that the spinal cord contains neuronal networks generating spinal PLRs; however, their efficacy is low, and supraspinal influences substantially contribute to generation of PLRs^4^,^5^. Recently, populations of spinal interneurons contributing to generation of PLRs have been characterized^6^. It was suggested that PLRs in intact animals substantially contribute to postural corrections^3^,^4^. Spinalization abolishes postural functions in quadrupeds, and postural control in spinal animals (including PLRs) does not recover with time^7^,^8^,^9^,^10^. After spinalization, spasticity (manifested in postural function as incorrect motor responses to posture-related sensory signals) gradually develops, suggesting plastic changes in the spinal postural networks^10^.

An immediate reaction to spinalization is “spinal shock”, characterized by a dramatic reduction of extensor tone and most spinal reflexes including PLRs^10^,^11^,^12^,^13^. The main reason for spinal shock is a loss of supraspinal influences on spinal networks^13^,^14^. It was demonstrated that one of the factors responsible for the reduced efficacy of spinal reflexes after spinalization is a decrease in the excitability of spinal motoneurons^15^,^16^,^17^. Recently, by using the method of “reversible spinalization” (a temporal cold block of signal transmission in spinal pathways) we have demonstrated that another factor is a decrease in the activity of most spinal interneurons, including PLR-related neurons^14^. However, the method of reversible spinalization did not allow for characterizing in detail the spinal postural networks at the acute stage of spinalization since the state of functional spinalization was maintained only for a few minutes (to prevent damage of the nervous tissue by cooling).

The overall goal of our current research is to reveal the changes in spinal postural networks that may underlie the development of spasticity^18^. The aim of the present study was to characterize in detail the starting point for these changes, that is the state of the spinal postural networks at the acute stage of spinalization. For this purpose, the responses of interneurons from L5 to stimulation that evoked PLRs before spinalization were recorded in decerebrate rabbits spinalized at T12. The results were compared with control data obtained in our previous study^6^.

A brief account of this study has been published in abstract form^19^.

## Results

### Effect of spinalization on PLRs

**Figure 1:**
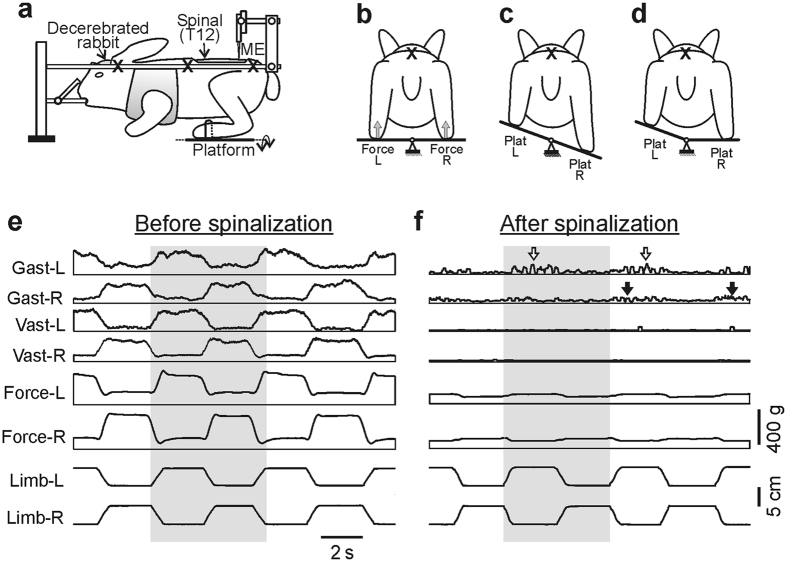
Experimental design and effect of acute spinalization on PLRs. (**a**) The decerebrate rabbit was fixed in a rigid frame (points of fixation are indicated by “X”). The hind limbs were positioned on a platform (**b**). The whole platform (**c**) or its left or right part (Plat L and Plat R in **d**) could be periodically tilted, causing flexion-extension movements of the two limbs (in anti-phase) or one of them, respectively. These movements were monitored by mechanical sensors (Limb-L and Limb-R, respectively). The contact forces under the left (L) and right (R) limbs were measured by the force sensors (Force-L and Force-R in **b**). After acute spinalization (Spinal in **a**), activity of spinal neurons from L5 was recorded by means of the microelectrode (ME). (**e**) Reflex electromyographic (EMG) and force responses to flexion/extension anti-phase movements of the hind limbs before spinalization. EMGs of m. *gastrocnemius lateralis* (Gast) and m. *vastus lateralis* (Vast) were recorded. (**f**) Reflex responses (in the same rabbit as in **e**) after spinalization at T12. White and black arrows indicate, respectively, residual correctly phased (in relation to the platform tilts) and incorrectly phased responses in Gast muscles.

Before spinalization, in all preparations, the whole platform tilts (Fig. 1c) evoked PLRs similar to those described in detail in our earlier studies^4^,^20^. They included activation of extensors in the flexing limb and an increase in its contact force, as well as inactivation of extensors in the extending limb and a decrease in its contact force, as illustrated in Fig. 1e. A separate tilt of the left or right platform evoked PLRs mainly in the ipsilateral limb (not illustrated). As shown in Fig. 1f, spinalization resulted in almost complete abolition of PLRs. The average value of the force response was 4–5 times smaller than that before spinalization [103 ± 8 g (N = 5, n = 67) and 539 ± 115 g (N = 5, n = 58), respectively, *P* < 0.0001] and did not differ statistically from the passive force measured after sacrificing the animal (100 ± 15 g, N = 5, n = 25, *P* = 0.851). The EMG responses were absent in 81% of cases, and weak residual responses were observed in 19% of cases; 8% of them were caused not by the limb flexion (as before spinalization), but by the limb extension and in 3% of cases the muscle responded both to the limb flexion and to its extension (N = 5, n = 67). Similar effects on force and EMG responses were observed in our previous experiments with acute spinalization^4^ and with reversible spinalization^14^.

### Effect of spinalization on spinal neurons

**Figure 2:**
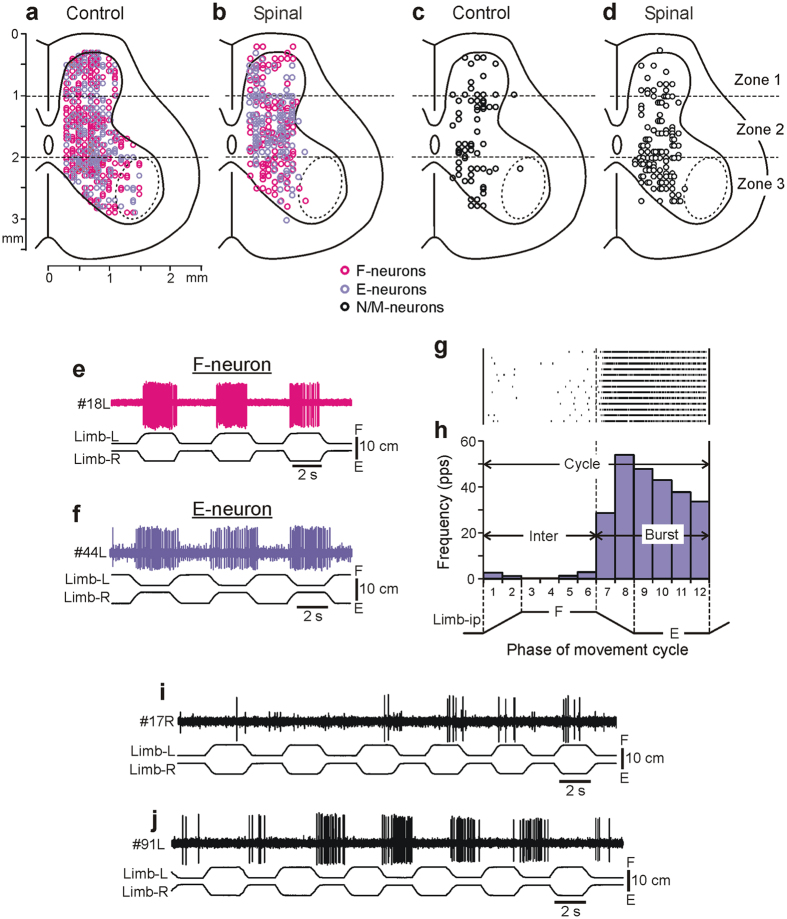
Classification of spinal neurons. (**a**,**b**) Position of all F- and E-neurons on the cross-section of the spinal cord recorded in control (**a**, *n* = 435) and in spinal rabbits (**b**, *n* = 249). (**c**,**d**) Position of all non-modulated neurons on the cross-section of the spinal cord recorded in control (**c**, n = 64) and in spinal rabbits (**d**, n = 121). The area of motor nuclei is indicated by a dotted line. Three zones of the grey matter are shown: the dorsal (1), intermediate (2) and ventral (3) ones. (**e**,**f**) An example of a neuron activated with flexion of the ipsilateral limb (F-neuron, **e**) and a neuron activated with its extension (E-neuron, **f**). (**g**) A raster of responses of E-neuron in 13 sequential movement cycles of the ipsilateral limb. (**h**) A histogram of spike activity (for the neuron shown in **f**) in different phases (1–12) of the cycle of movement (**F**, flexion; E, extension) of the ipsilateral limb (Limb-ip). The halves of the cycle with higher (**E**, bins 7–12) and lower (**F**, bins 1–6) neuronal activity were designated as “Burst” and “Interburst” periods, respectively. (**i**,**j**) Examples of neurons with inconsistent response to tilt.

In decerebrate rabbits subjected to acute spinalization at T12, 370 neurons in total were recorded during passive flexion/extension limb movements caused by periodical tilts of the whole platform. The majority of these neurons were recorded outside the area of motor nuclei (Fig. 2a–d, dotted line) and thus were considered as putative interneurons. 67% of all recorded neurons were modulated, whereas 33% were not modulated by platform tilts. According to the phase of neuronal response in the movement cycle of the ipsilateral limb, all modulated neurons were divided into two groups. F-neurons (33% of all recorded neurons) had a higher frequency in the flexion phase than in the extension phase (as the neuron in Fig. 2e), whereas E-neurons (34% of all recorded neurons) had a higher frequency in the extension phase (as the neuron in Fig. 2f). Neurons of both F and E groups, as well as non-modulated neurons, were found in each individual rabbit. In 9% of modulated neurons we observed inconsistent modulation (i.e., a neuron responded not in all tilt cycles as the neuron in Fig. 2i) or had a large variability in the burst duration or frequency (as the neuron in Fig. 2j). Such neurons were never observed in control.

To reveal the effects of acute spinalization on spinal postural networks, the data obtained in the present study were compared to control data from our previous study on decerebrate rabbits with intact spinal cord^6^.

#### Distribution of F- and E-neurons

Spinalization caused a significant decrease in the proportion of modulated neurons (67% vs 82% in control, χ^2^ test, *P* < 0.0001). Figure 2a,b shows location (on the cross-section of segment L5) of all individual F- and E-neurons recorded in rabbits with an intact spinal cord (Control^6^) and in spinal rabbits (Spinal). In control, F-neurons outnumbered E-neurons. Spinalization resulted in a significant decrease of the F/E ratio, from 1.34 to 0.96 (χ^2^ test, *P* < 0.05).

Both in control (Fig. 2a) and in spinal rabbits (Fig. 2b), F- and E-neurons were widely distributed over the cross-section of the grey matter of the spinal cord. To characterize changes in their distribution caused by spinalization, the cross-section was divided into three zones (1–3), and the number of F- and E-neurons in each of the zones was calculated in control and after spinalization. In control, F-neurons were more numerous in any of the zones 1–3. Acute spinalization resulted in a significant decrease in the F/E ratio in zone 2 (from 1.49 to 0.63; χ^2^ test, *P* < 0.001) and insignificant changes in zones 1 and 3 (from 1.27 to 1.00, and from 1.26 to 2.26, χ^2^ test, *P *> 0.05 and *P *> 0.05, respectively).

#### Activity of F-neurons

The effects of spinalization on different parameters of the activity of F-neurons are shown in Table 1. One can see that the mean frequency and the depth of modulation averaged over all F-neurons (All in Table 1) significantly decreased. This was caused mainly by a significant decrease in the burst frequency, whereas the change in the interburst frequency was not significant. A similar tendency was observed in the changes of activity of F-neurons located in each of three zones of the grey matter (Zones 1–3 in Table 1).

**Figure 3:**
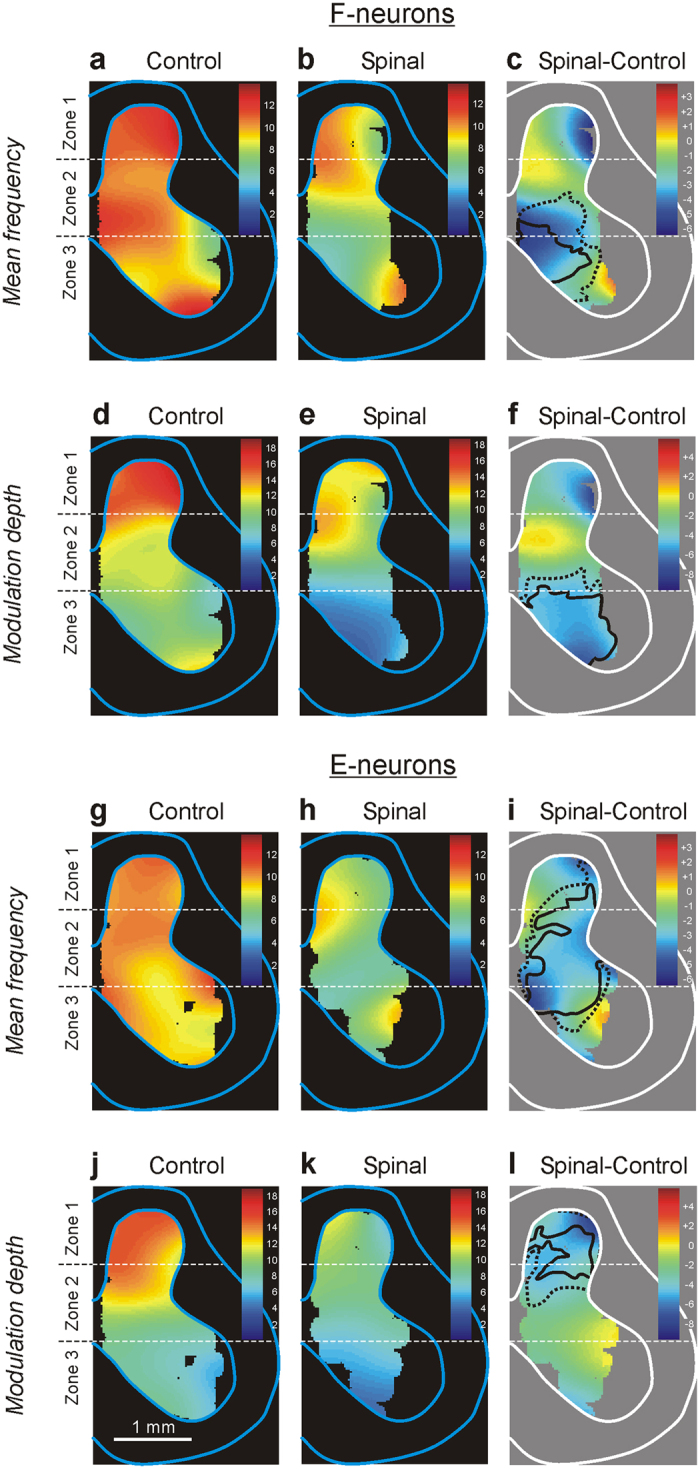
Effects of spinalization on the activity of F- and E-neurons during tilts of the whole platform. (**a–f**) Averaged distributions of the mean frequency (**a**,**b**) and of the depth of modulation (**d**,**e**) of F-neurons on the cross-section of the spinal cord in control (**a**,**d**) and after acute spinalization (**b**,**e**). (**c**,**f**) The difference between the two distributions (subtraction of Control from Spinal). (**g–l**) Averaged distributions of the mean frequency of E-neurons (**g**,**h**) and the depth of modulation (**j**,**k**) on the cross-section of the spinal cord in control (**g**,**j**) and after acute spinalization (**h**,**k**). (**i**,**l**) The difference between the two distributions (subtraction of Control from Spinal). The mean values are presented as heatmaps (see Materials and Methods). The areas of significant changes of the local mean frequency are delimited by a solid line (t-test, *p* < 0.01) and a hatched line (t-test, *p* < 0.05).

To reveal the effect of spinalization on the activity of local populations of F-neurons in different areas of the grey matter, we used the averaged distribution of two parameters of the neuronal activity – the mean frequency and the depth of modulation – across the gray matter of the spinal cord (heatmaps, see Methods). Figure 3a,b shows heatmaps for the mean frequency of F-neurons in control and after spinalization, respectively. One can see that spinalization caused a reduction in the activity of neuronal populations in different areas of the grey matter. This reduction was maximal in the neuronal populations located in the ventral half of zone 2, the medial part of zone 3, and the lateral part of the dorsal horn, as shown by subtracting Control from Spinal in Fig. 3c. However, this decrease in the mean frequency by 2–6 pps was statistically significant only in zone 2 and zone 3 (delineated by solid and hatched lines for *P* = 0.01 and *P* = 0.05, respectively, Fig. 3c).

Figure 3d–f shows heatmaps for the depth of modulation of F-neurons in control (d) and after spinalization (e), as well as the changes in the depth of modulation after spinalization (f). One can see that spinalization caused a significant reduction of the depth of modulation in the neuronal populations located in the ventro-medial part of the ventral horn. Thus, the areas in which neuronal populations exhibited a maximal reduction in the depth of modulation and in the mean frequency considerably overlapped with zone 3. An almost 30% decrease in the mean frequency and burst frequency, and a two-fold decrease in the depth of modulation were observed in neurons located in zone 3 (Table 1).

#### Activity of E-neurons

The effects of spinalization on the activity of E-neurons are shown in Table 1. These effects on all population of E-neurons were basically similar to those found in F-neurons – after spinalization there was a significant decrease in all parameters of neuronal activity as compared to control. However, there were some differences when comparing the effects of spinalization on E- and F-neurons located in each of the three zones (Table 1). In E-neurons from zones 1 and 2, the decrease in most parameters was statistically significant, while in zone 3 it was insignificant. By contrast, in F-neurons the decrease in most parameters was significant in zone 3, but insignificant in zones 1 and 2.

Figure 3g,h,j,k, shows heatmaps for the mean frequency and the depth of modulation of E-neurons in control and after spinalization, respectively. One can see that spinalization caused a significant reduction in the mean frequency of E-neurons located in the dorsal part of the ventral horn, in the intermediate area and in the ventro-lateral part of the dorsal horn (Fig. 3i).

Spinalization caused a significant reduction in the depth of modulation in the populations of E-neurons located in the dorsal horn, whereas E-neurons in the intermediate area and in the ventral horn were almost not affected (Fig. 3l). Thus, in contrast to F-neurons, the local populations of E-neurons exhibiting a significant reduction in the mean frequency, and the populations exhibiting a significant reduction in the depth of modulation had mostly different location (compare Fig. 3c,f,i,l, respectively).

#### Activity of non-modulated neurons

In the present study, 121 neurons unmodulated by the whole platform tilts were recorded. They constituted 33% of all neurons *vs* 18% (64 out of 360 neurons) in control. This increase was significant (χ^2^ test, P < 0.0001). Both, in control and in spinal animals, non-modulated neurons were found in all three zones of the grey matter (Fig. 2c,d). Their distribution across the gray matter was not affected by spinalization (χ^2^ test for the number of non-modulated neurons in zones 1–3 in intact animals and after spinalization, *P *> 0.05). The mean frequency averaged over all non-modulated neurons was significantly decreased (All in Table 1). Substantial reduction in activity was observed in unmodulated neurons located in each of three zones of the grey matter (Table 1).

### Processing of tilt-related sensory information

Neuronal responses to tilts are driven by the somatosensory input from the limbs. To characterize the effects of spinalization on the processing of tilt-related sensory information, in 211 neurons we recorded responses not only to tilts of the whole platform, but also to tilts of its right or left part (Fig. 1d), and compared the obtained results to control.

#### Sources of modulation of F- and E-neurons

By tilting either the left or right platform alone, we determined the sources of sensory input to individual neurons. As in control^6^, in spinal animals four types of neurons differing in the combination of tilt-related somatosensory inputs from the ipsilateral and contralateral limb, as well as in the direction of imposed movement (limb flexion or extension) activating the neuron, were found. Type 1 neurons were activated by ipsi-limb flexion or extension; Type 2, by contra-limb flexion or extension; Type 3, by ipsi-limb flexion and contra-limb extension, or ipsi-limb extension and contra-limb flexion; Type 4, by ipsi-limb and contra-limb flexion, or ipsi-limb and contra-limb extension.

**Figure 4:**
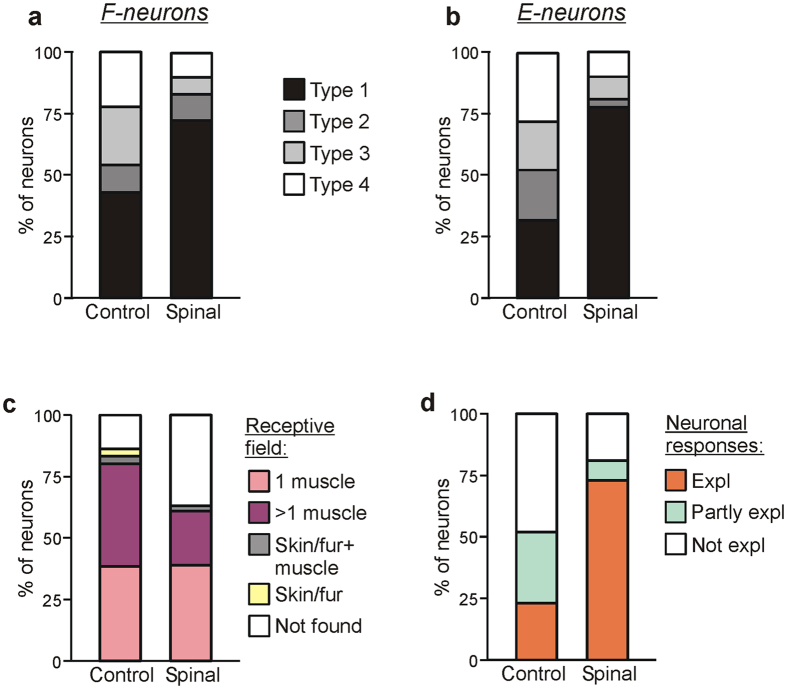
Effect of spinalization on sources of modulation and on receptive fields of F- and E-neurons. (**a**,**b**) Percentage of F- and E-neurons (**a** and **b**, respectively) receiving different combinations of tilt-related somatosensory inputs from the limbs (Types 1–4) in control and after spinalization. See text for explanation. (**c**) Proportion of neurons receiving sensory inputs from different sources, i.e., from receptors of only one muscle (1 muscle), from receptors of more than one muscle (>1 muscle), from cutaneous and muscle receptors (Skin/fur + muscle), from cutaneous receptors only (Skin/fur), and with no receptive field found (Not found) in rabbits with an intact spinal cord (Control) and in spinal rabbits (Spinal). See text for explanation. (**d**) Proportion of neurons in which response to tilts could be completely explained (Expl), partly explained (Partly expl) and could not be explained (Not expl) by input from their receptive field, in rabbits with an intact spinal cord (Control) and in spinal rabbits (Spinal).

Figure 4a,b shows the relative number of neurons of different types in control and after spinalization, separately for F-neurons (a) and E-neurons (b). After spinalization, about a two-fold increase in the relative number of Type 1 neurons (73% *vs* 43% in control for F-neurons, and 78% *vs* 32% in control for E-neurons) was observed. Correspondingly, the relative number of neurons receiving input from the contralateral limb (Types 2–4), decreased (28% *vs* 56% in control for F-neurons, and 22% *vs* 69% in control for E-neurons). These changes in proportions of Type 1 neurons and Types 2, 3 and 4 neurons were significant both for F- and E-groups (χ^2^ test for the number of neurons receiving or not receiving inputs from the contralateral limb, in intact animals and after spinalization, *P* < 0.0001).

#### Efficacy of sensory inputs to F- and E-neurons from different limbs

##### Inputs to F-neurons

Table 2 shows the effect of spinalization on the efficacy of sensory inputs to F-neurons and to E-neurons from the ipsilateral (Ipsi-tilts) and from the contralateral limb (Contra-tilts). In control, all parameters of neuronal activity (except for the interburst frequency), averaged over all F-neurons were much larger during tilts of the ipsilateral platform than during tilts of the contralateral platform. Spinalization caused a significant decrease of these parameters in both cases. A similar tendency was observed in the changes of activity of F-neurons located in each of three zones of the grey matter.

**Figure 5:**
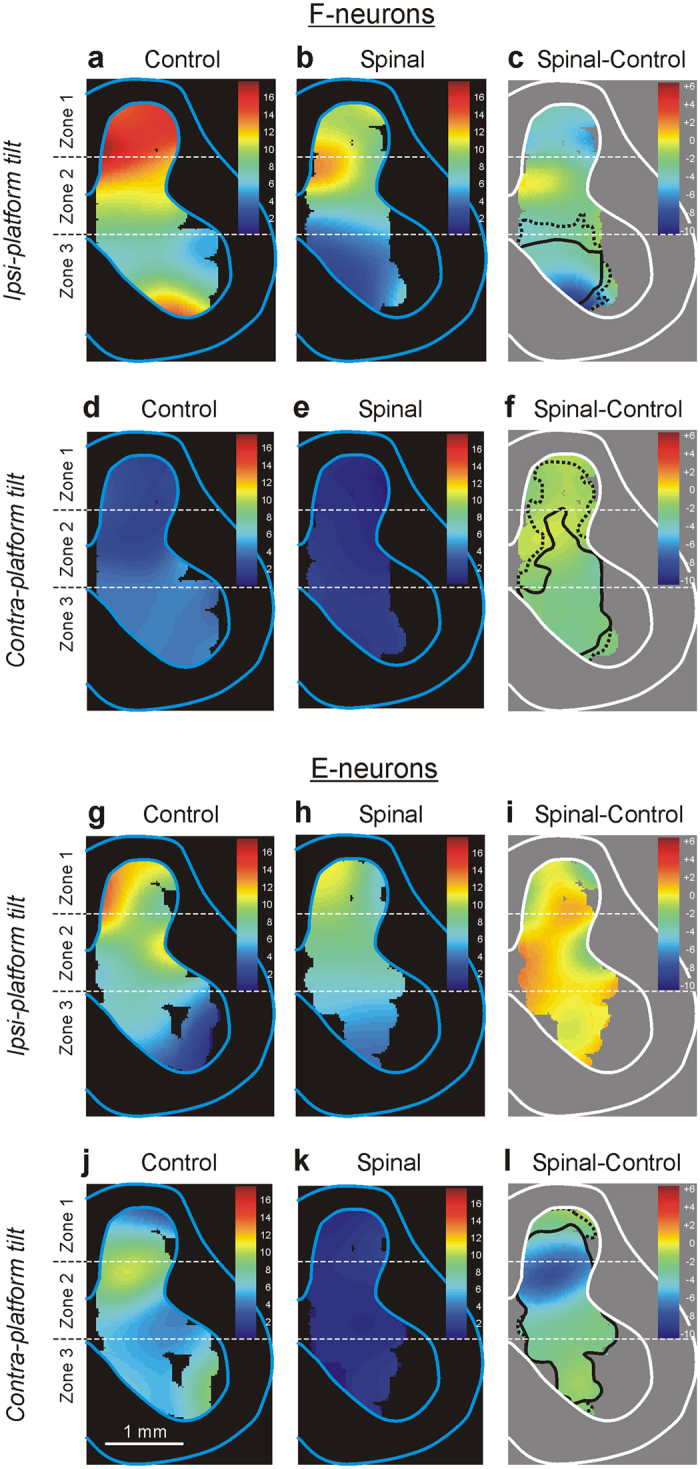
Effects of spinalization on the efficacy of tilt-related sensory inputs from the ipsilateral and contralateral limbs to F- and to E-neurons. (**a,b,d,e)** Heatmaps for the depth of modulation of F-neurons recorded during tilts of the ipsilateral platform only (**a,b**) and contralateral platform only (**d,e**) in control (**a,d**) and in acute spinal rabbits (**b,e**). The difference between the two distributions (that is subtraction of Control from Spinal) during tilts of the ipsilateral platform only (**c**) and contralateral platform only (**f**). (**g,h,j,k**) Heatmaps for the depth of modulation of E-neurons recorded during tilts of the ipsilateral platform only (**g,h**) and contralateral platform only (**j,k**) in control (**g,j**) and in acute spinal rabbits (**h,k**). The difference between the two distributions (that is subtraction of Control from Spinal) during tilts of the ipsilateral platform only (**i**) and contralateral platform only (**l**). Designations are the same as in Fig. 3.

The heatmaps for the depth of modulation of F-neurons (Fig. 5a–f) show that spinalization resulted in a reduction in the efficacy of input from the ipsilateral limb to the neuronal populations across the whole gray matter (compare a and b). However, this reduction was statistically significant only in the populations of F-neurons located in the ventral horn (Fig. 5c). In this area, which mostly coincides with zone 3, a 40% decrease in the mean frequency and burst frequency, and more than two-fold decrease in the depth of modulation were observed (Table 2).

Spinalization caused an almost complete disappearance of the input from the contralateral limb: the depth of modulation in local populations of F-neurons decreased from 2–5 pps in control (Fig. 5d) to 0–1 pps after spinalization (Fig. 5e). These changes in the depth of modulation were significant across almost entire grey matter, but were most pronounced in the ventral horn (Fig. 5f). In this area (which coincides with zone 3), about 40% decrease in all parameters of activity was observed (Table 2). Thus, spinalization caused a dramatic decrease in the efficacy of tilt-related sensory input from both limbs to F-neurons located in zone 3. One can suggest that a decrease in sensory input from both limbs is responsible for a decrease in response of F-neurons to whole platform tilts after spinalization.

##### Inputs to E-neurons

In contrast to F-neurons, in control, almost all parameters of neuronal activity averaged over all E-neurons were similar during tilts of the ipsilateral platform and during tilts of the contralateral platform (Table 2). As in F-neurons, spinalization caused a decrease in most parameters of activity of E-neurons. However, in contrast to F-neurons, it produced much stronger effect on the activity of E-neurons during tilts of the contralateral platform than during tilts of the ipsilateral platform (Table 2).

The heatmaps for the depth of modulation of E-neurons (Fig. 5g–l) show that, in contrast to F-neurons (Fig. 5c), in E-neurons spinalization caused no significant reduction in the efficacy of input from the ipsilateral limb (Fig. 5i). However, as in F-neurons (Fig. 5e), it caused almost complete disappearance of the contralateral input, since the depth of modulation decreased from 4–11 pps in control (Fig. 5j) to 0–1 pps (Fig. 5k). These changes in the depth of modulation were significant across the entire grey matter (Fig. 5l). A significant (40–50%) decrease in other parameters of activity (the mean frequency and the burst frequency) of E-neurons located in each of three zones was also observed during Contra-platform tilts (Table 2). Thus, in contrast to F-neurons, mainly a decrease in efficacy of tilt-related sensory input from contralateral limb is responsible for a decrease in response of E-neurons to whole platform tilts after spinalization.

#### Relation between responses to tilts and receptive fields of neurons

After spinalization, somatosensory receptive fields were found in 120 out of 196 tested modulated neurons. The proportion of such neurons was smaller after spinalization (63%) than in control (86%) (Fig. 4c). As in control^6^, in the majority of neurons (119 out of 196, 61%) the receptive fields were “deep”: the neurons responded to palpation of muscles or to movements of joints, but not to stimulation of the fur or skin alone. The neurons with responses to stimulation of fur or/and skin were in minority (5 out of 196, 2%). Spinalization resulted in an almost two-fold decrease in the percentage of neurons with multiple (from more than 1 muscle) deep receptive fields (22% *vs* 42% in control) and a more than two-fold increase in the proportion of neurons in which receptive fields were not found (37% *vs* 14% in control). These changes were statistically significant (χ^2^ test for the number of neurons with different receptive fields in intact animals and after spinalization, *P* < 0.0001).

For 98 modulated neurons with deep receptive fields, we compared responses of a neuron to tilts with afferent signals that the neuron presumably receives from its receptive field during tilts. One could expect that tilt of the platform would activate stretch and load receptors in extensors of the flexing limb, and those in flexors of the extending limb.

It was found that in spinal rabbits, the majority of neurons (70 out of 98, 73%) had “corresponding” receptive fields, i.e., their response to tilts could be fully explained by receptive field inputs (Expl in Fig. 4d, Spinal). Eight neurons (8%) had afferent inputs that could be responsible for their reaction to tilts, but they also had mismatching inputs, for example, excitatory inputs from the antagonistic muscles of one limb (Partly expl in Fig. 4d, Spinal). Finally, modulation of 18 neurons (19%) could not be explained by input from the receptive field (Not expl in Fig. 4d, Spinal). Figure 4d also shows the proportions of neurons with different role of receptive field input in their modulation in control animals. One can see that spinalization caused more than three-fold increase in the relative number of neurons in which receptive field input could explain the response to tilts (73% *vs* 23% in control), and the corresponding decrease in the relative number of neurons in which response could be partly explained (8% *vs* 29% in control) or not explained (19% *vs* 48% in control) by input from the receptive field.

## Discussion

In the present study we characterized the activity of spinal neurons (putative interneurons) of postural networks, as well as the processing of posture-related sensory information in acutely spinalized rabbits. Comparison of these data with the data obtained in our previous study^6^ on rabbits with undamaged spinal cord (control) allowed us to characterize the effects of spinalization on these networks.

As in control, all recorded neurons in acutely spinalized rabbits, in accordance with their responses to tilts, were divided into three groups: F-neurons, E-neurons and non-modulated neurons. The proportion of non-modulated neurons in spinal rabbits was larger than in control (33% *vs* 18%). This could be explained by the fact that a part of modulated neurons, after elimination of supraspinal drive, became non-modulated, as demonstrated with the method of reversible spinalization^14^.

It was suggested that in animals with an intact spinal cord, F- and E-neurons contribute to generation of PLRs^6^,^21^. Since elimination of supraspinal drive did not affect the phase of modulation in most F- and E-neurons^14^, one can suggest that recorded in the present study F- and E-neurons are elements of spinal postural networks generating spinal PLRs. However, in spinal animals these networks require additional activation for their functioning^4^.

F-neurons and E-neurons are not homogeneous groups; they possibly include segmental and propriospinal interneurons, as well as neurons of ascending tracts^22^,^23^,^24^,^25^,^26^. Since in control F- and E-neurons are modulated in-phase and in anti-phase with extensor motoneurons, it was suggested that at least some of them are pre-motor interneurons that excite and inhibit extensor motoneurons, respectively^6^. Such pre-motor interneurons with somatosensory inputs from the limb were found in the lumbosacral spinal cord^27^,^28^,^29^,^30^.

We found that spinalization affected a relative size of F- and E-groups of neurons, as well as their distribution in the grey matter (Table 1). The increase in the relative number of E-neurons could be explained by a bias toward F-neurons in the population of modulated neurons completely inactivated by spinalization, and by uneven distribution of such neurons between zones 1–3. The existence of modulated neurons completely inactivated by spinalization, was demonstrated^14^.

We found that most parameters of activity of spinal neurons (mean frequency, burst frequency, and depth of modulation) in spinal rabbits were, on average, significantly lower than in the rabbits with intact spinal cord (Table 1). This decrease in activity of F- and E-neurons could be caused by three factors: *first*, by a decrease in excitability of spinal interneurons after elimination of supraspinal drive; *second*, by a decrease in efficacy of sensory input from limb mechanoreceptors due to distortions in processing of this input; *third*, by a decrease in the value of sensory input. The latter factor was due to a dramatic reduction in the forces developed by extensor muscles (Fig. 1e,f) and monitored by load receptors, as well as due to inactivation of gamma-motoneurons (which receive significant supraspinal influences^31^,^32^,^33^, leading to a decrease in signals from muscle spindles.

We found that spinalization affected differently activity of F- and E-neurons located in different zones of the grey matter. The largest decrease of most parameters of activity was observed within zone 3 in F-neurons, and within zones 1 and 2 in E-neurons (Table 1).

To localize the population of neurons most affected by spinalization, we used heatmaps and found an area (spanning the ventral half of zone 2 and dorsal half of zone 3), in which a significant decrease in the mean frequency of both F- and E-neurons (Fig. 3c,i, respectively) and a significant decrease in the modulation depth of F-neurons (Fig. 3f) were observed. In the same area, most neurons, almost completely inactivated during reversible spinalization, were found^14^. The majority of these neurons were F-neurons. One can suggest that F-neurons located in this area are excitatory pre-motor interneurons responsible for activation of extensor motoneurons during PLRs, and their inactivation in spinal rabbits contributes substantially to loss of PLRs. It was demonstrated that some neurons in this area produce excitation of extensor motoneurons^34^ .

It is noteworthy that the maximal reduction in the population activity of putative interneurons of postural networks after spinalization was less than two-fold (All in Table 1). By contrast, the reduction in EMGs and force responses to tilts was much larger (compare Fig. 1e,f; see also^4^). However, one should take into account that elimination of supraspinal drive caused by reversible spinalization^14^ resulted in a substantial decrease (by 38%) in the relative number of neurons with activity modulated by tilts. One can conclude that, among other factors, both a decrease in the number of modulated neurons and a reduction in the activity of still modulated neurons, as well as a decrease in the excitability of spinal motoneurons due to the loss of direct supraspinal influences^15^,^16^, contribute to PLRs loss.

The present study has shown that spinalization affected the contribution of sensory inputs from the ipsilateral and contralateral limbs to modulation of F- and E-neurons. We have found an almost two-fold increase in the proportion of neurons modulated by sensory input from the ipsilateral limb (Type 1), and a corresponding decrease in the proportion of neurons with a contribution of input from the contralateral limb (Types 2–4, see Fig. 4a,b). This was caused by a significant reduction in the efficacy of tilt-related sensory inputs from the contralateral limb to both F- and E-neurons across the entire gray matter (Fig. 5d–f,j–l, respectively). Most likely, commissural interneurons transmitting signals from the contralateral limb are inactivated by acute spinalization. Such commissural neurons, with sensory input from the limb, and inputs from supraspinal structures, have been described^35^. Many of these neurons are located in the ventro-medial areas, which were most affected by spinalization (Fig. 3c,i).

Spinalization affected differently the efficacy of sensory inputs from the ipsilateral limb to F- and E-neurons. The efficacy of these inputs to E-neurons remained almost unchanged (Fig. 5g–i), but it was significantly decreased for F-neurons located in zone 3 (Fig. 5a–c). Since there were differences in the strength of tilt-related sensory inputs from the ipsilateral and contralateral limb to F- and E-neurons in control (compare Fig. 5a,d,g,j, respectively), one can suggest that a decrease in response of F- and E-neurons to whole platform tilts after spinalization was caused mainly by a decrease in input from the ipsilateral limb to F-neurons, and by a decrease in input from the contralateral limb to E-neurons.

We have found that in spinal rabbits, responses to tilts in most neurons could be explained by sensory signals from their receptive fields (Fig. 4d, Spinal). By contrast, in control, such neurons constituted a minority (Fig. 4d, Control). One can suggest that in control, when platform tilts simultaneously activate receptors of several muscles, signals from these receptors are processed by a specific network, which differs from the network processing signals from receptors of individual muscles when they are activated alone.

Immediate consequence of spinalization is spinal shock, defined as a combination of muscular paralysis, hypotonus, and abolition of most spinal reflexes^11^,^12^,^13^. In the present study the state of spinal networks at spinal shock condition has been characterized. Spinal shock is followed by gradually developing spasticity, defined as a combination of abnormal reflex responsiveness, clonus, and hypertonus^12^,^36^,^37^. It was suggested that one likely reason for spasticity is gradual reorganization of spinal reflex pathways induced by spinal cord injury^24^,^38^,^39^,^40^,^41^,^42^,^43^,^44^. The data obtained in the present study represent a starting point for these changes. We have demonstrated that some reorganization of reflex pathways underlying appearance of incorrectly phased EMG responses to tilts, takes place as early as in the acute state.

To conclude, in the present study the activity of spinal neurons of postural networks in acutely spinalized mammals have been characterized for the first time. A significant reduction in the activity of these neurons located in particular areas of the grey matter was found. Specific changes in the efficacy of posture-related sensory inputs to different neuronal groups have been demonstrated. These distortions in the operation of postural networks contribute to postural deficits observed after spinalization. Characterization of neuronal activity in acute spinal animals provides a starting point for studies of long-term plastic changes in the spinal networks following spinalization.

## Methods

Experiments (N = 5) were carried out on adult New Zealand rabbits (weighing 2.5–3.0 kg). All experiments were conducted in accordance with NIH guidelines and were approved by the local ethical committee (Norra Djurförsöksetiska Nämden) in Stockholm. The data obtained in the present study were compared with the control data taken from the database of our previous study^6^. The experimental subjects, as well as all methods (except for spinalization) used in the present study were similar to those used in the control study^6^. They are briefly described below. The control data for F- and E-neurons were published earlier^6^. The control data for non-modulated neurons recorded in 15 out of 20 experiments of the control study were not published previously, and are presented in this paper.

### Surgical procedures

The animal was injected with propofol (average dose, 10 mg/kg, administrated intravenously) for induction of anaesthesia, which was continued on isoflurane (1.5–2.5%) delivered in O_2_. The trachea was cannulated. The spinal cord was exposed by laminectomy at T11-L1 (for the subsequent spinalization), as well as at L5 (for recording of neurons). At T12, the dura mater was removed for subsequent spinalization. Small holes (~1 mm^2^) were made in the dura mater at L5 to insert the recording microelectrode. Bipolar EMG electrodes were inserted bilaterally into gastrocnemius lateralis (ankle extensor) and vastus lateralis (knee extensor).

The animal was then decerebrated at the precollicular-postmammillary level^20^. After decerebration, the anaesthesia was discontinued. In 1 h after cessation of anesthesia, to examine the functional state of the preparation, PLRs were tested. The spinal cord was completely transected using micro scissors. During the experiment, the rectal temperature and mean blood pressure of the animal were continuously monitored and were kept at 37–38°C and at greater than 90 mmHg, respectively. Recordings of neurons began about 30–40 min after spinalization.

### Experimental design

The experimental design is shown in Fig. 1a and was similar to that described earlier^4^,^6^,^14^,^21^. In short, the head and vertebral column were rigidly fixed; the forelimbs were suspended in a hammock. The hind limbs were positioned on the horizontal platform, with the limb configuration and inter-feet distance similar to that observed in freely standing rabbits^2^. The platform as a whole, or its left and right part separately, could be tilted periodically by rotation around the medial axis (Fig. 1b–d) between two positions: 20° to the left (L) and 20° to the right (R). A time trajectory of tilting the platform and, therefore, a time trajectory of foot displacement was trapezoidal with a period of ∼6 s (Fig. 1e,f); transition between extreme positions lasted for ∼1 s, and each extreme position was maintained for ∼2 s. Since the vertebrate column and pelvis were fixed, tilts of the platform led to flexion/extension movements at the hip, knee, and ankle joints, with the magnitude of ∼10° and close-to-vertical displacements of the distal point of the limb with amplitude ∼5 cm. The tilt angle of each platform was monitored by mechanical sensors, scaled and recorded as the vertical foot position. The contact forces under the limbs were measured by means of force sensors (Force in Fig. 1b).

### Recording of neurons and data analysis

In five rabbits 370 neurons were recorded (n = 122, 120, 110, 13, 5 in individual animals). Neurons were recorded extracellularly from the spinal segment L5 by means of commercially available varnish-insulated tungsten electrodes (75 μm shaft diameter; FHC, Bowdoin, ME). The impedance of the electrodes was 4–7 MΩ. We tended to explore systematically the whole cross-section of the grey matter except for the area of motor nuclei indicated by the dotted line in Fig. 2a,b 45. We performed recordings of individual neurons with spikes of different amplitudes, as well as simultaneous recordings of several neurons with clearly different spike shape or amplitude. The lateral and vertical coordinates of each neuron were marked on the map of the spinal cord cross-section^14^,^21^,^46^.

Activity of individual neurons, along with EMGs and ground reaction forces, was recorded during alternating flexion/extension movements of the limbs caused by whole platform tilts in many (up to 10) sequential tilt cycles. To reveal tilt-related somatosensory inputs from the left and right limbs to individual neurons, the majority of neurons were also recorded during separate tilts of the right and left platform (Fig. 1d).

Peripheral receptive fields were examined in some of the neurons. Stimuli included light brushing of the hairs, light tapping of hair skin, firm tapping on and palpation of muscle bellies (flexors and extensors of ankle, knee and hip, and abductors and adductors of hip) and tendons, pinching the skin with fingers, and in a few cases manual movements of joints. If a cutaneous input was observed then the responses from the underlying muscles were not taken into account. Stimuli that could potentially activate nociceptors (i.e. pinching or poking with sharp instruments) were not used. Complete testing of an individual neuron usually took 3–5 minutes.

Signals from the microelectrode (neuronal activity), from the EMG electrodes, and from the position and force sensors were amplified, digitised with a sampling frequency of 30 kHz (neuron), 5 kHz (EMGs) and 1 kHz (sensors), and recorded on a computer disk using the data acquisition and analysis system (Power-1401/Spike2, Cambridge Electronic Design, Cambridge, UK). This system was then used for offline waveform analysis to discriminate and identify the spikes of a single neuron using the waveform-matching algorithm. Only neurons with a stable spike shape were used for analysis.

When processing the recorded data, we considered the activity of all individual neurons in the movement cycle of the ipsilateral limb since the phase of modulation in the majority of neurons was determined by the tilt-related sensory input from the ipsilateral limb. The onset of the ipsilateral limb flexion was taken as the cycle onset. When only the contralateral platform was tilted, the cycle started at the onset of extension of the contralateral limb.

For each individual neuron, a raster of activity in sequential movement cycles was obtained. The raster for one of the neurons is shown in Fig. 2g. The cycle was divided into 12 bins, and the onset of flexion of the ipsilateral limb was taken as the cycle onset. Bins 1–2 corresponded to flexion of the limb; bins 3–6, to maintenance of the flexed position; bins 7–8, to extension of the limb; and bins 9–12, to maintenance of the extended position. The firing frequency in each bin was calculated and averaged over the identical bins in all cycles at a given condition, and the phase histogram was generated (Fig. 2h). The mean frequency during flexion of ipsilateral limb (bins 1–6) and that during extension (bins 7–12) was compared. The larger and the smaller values were named the burst frequency (F_burst_) and the interburst frequency (F_inter_), respectively. The neuron was considered to be modulated by tilts if the difference between the mean burst frequency and mean interburst frequency was statistically significant (two-tailed Student’s *t*-test, *P* < 0.05). Then we calculated the mean frequency (average value over bins 1–12) and the depth of modulation M = F_burst_ − F_inter_.

Since it was shown that the majority of PLR-related neurons preserved the phase of their response to platform tilts during reversible spinalization^14^, we used the same classification of neurons as in our previous studies^6^,^14^,^21^: the neurons generating burst during flexion of the ipsi-limb (Fig. 2e) were termed F-neurons, and those generating burst during limb extension (Fig. 2f), E-neurons.

To characterize the effects of spinalization on the activity of local populations of neurons in different areas of the grey matter, “heatmaps” were generated for two parameters of neuronal activity – the mean frequency and the depth of modulation. In these maps, each value of a parameter was coded by a specific colour (see Figs 3,5). To calculate a value in the heatmap point with coordinates (*x*,*y*), we used measured values of the parameter for the neurons that were recorded in the vicinity of the point. The measured values were weighted depending on the distance *d* from (*x*,*y*) to the recording point (Gaussian weighting *w*(*d*) = *exp*(*−d*^*2*^*/D*^*2*^) with the spatial constant *D* = 0.4 mm). Similar heatmaps were built for the local changes of the corresponding parameters and for the *P*-value of the changes evaluated with the Student’s t-test (two-tailed). Areas of significant local changes (t-test, *P* = 0.01 and *P* = 0.05) were delimited by solid and hatched lines, correspondingly (e.g. Fig. 3c,f,i,l).

All quantitative data in this study are presented as mean ± s.e.m. Student’s t-test (two-tailed) was used to characterize the statistical significance when comparing different means; the significance level was set at *P* = 0.05. To evaluate the statistical significance of the effects of spinalization on the proportion of different functional groups of neurons, we used Pearson’s χ^2^ test; the significance level was set at *P* = 0.05.

### Histological procedures

At the end of experiment, reference electrolytic lesions were made in the spinal cord. The spinal cord was fixed with 10% formalin solution. Frozen sections of 30 μm thickness were cut in the region of recording. The tissue was stained for Nissl substance with Cresyl violet. Positions of recording sites were estimated in relation to the lesions.

## Additional Information

**How to cite this article**: Zelenin, P. V. *et al*. Effects of acute spinalization on neurons of postural networks. *Sci. Rep.* **6**, 27372; doi: 10.1038/srep27372 (2016).

## Figures and Tables

**Table 1:** Effects of spinalization on the activity of F-, E- and non-modulated neurons.

	**All**		**Zone 1**		**Zone 2**		**Zone 3**	
	**Control**	**Spinal**	**Control**	**Spinal**	**Control**	**Spinal**	**Control**	**Spinal**
**F-neurons**	n = 249	n = 122	n = 62	n = 30	n = 94	n = 49	n = 93	n = 43
F mean	10.6 ± 0.5	8.1 ± 0.6**	12.3 ± 0.9	9.9 ± 1.2	10.8 ± 1.0	8.2 ± 1.0	9.1 ± 0.7	6.4 ± 0.8*
F burst	16.5 ± 0.8	12.4 ± 0.9***	20.8 ± 1.5	16.4 ± 1.7	16.7 ± 1.5	13.0 ± 1.7	13.5 ± 1.0	8.7 ± 1.0**
F interburst	4.6 ± 0.4	3.7 ± 0.5	3.7 ± 0.6	3.5 ± 1.1	4.9 ± 0.7	3.4 ± 0.7	4.8 ± 0.6	4.2 ± 0.7
Mod depth	12.0 ± 0.7	8.7 ± 0.8**	17.1 ± 1.5	12.9 ± 1.5	11.8 ± 1.2	9.6 ± 1.5	8.7 ± 0.7	4.6 ± 0.6***
**E-neurons**	n = 186	n = 127	n = 49	n = 30	n = 63	n = 78	n = 74	n = 19
F mean	9.8 ± 0.5	7.2 ± 0.5***	10.4 ± 0.8	7.4 ± 0.7*	9.6 ± 0.9	6.9 ± 0.6*	9.6 ± 0.8	7.5 ± 1.5
F burst	15.1 ± 0.8	11.3 ± 0.7***	17.8 ± 1.3	12.6 ± 1.2**	15.0 ± 1.6	11.0 ± 1.0*	13.5 ± 1.0	10.4 ± 1.6
F interburst	4.5 ± 0.4	3.8 ± 0.4*	3.1 ± 0.7	2.9 ± 0.7	4.3 ± 0.6	2.8 ± 0.5	5.6 ± 0.7	4.6 ± 1.5
Mod depth	10.6 ± 0.7	8.2 ± 0.7*	14.7 ± 1.3	9.8 ± 1.4*	10.7 ± 1.6	8.1 ± 0.9	7.8 ± 0.6	5.7 ± 0.9
**N/M-neurons**	n = 64	n = 121	n = 14	n = 18	n = 27	n = 44	n = 23	n = 59
F mean	10.1 ± 1.1	6.1 ± 0.6**	10.6 ± 2.8	5.9 ± 1.3	9.0 ± 1.3	6.6 ± 1.0	11.1 ± 2.0	5.7 ± 0.7*

Designations: N/M-neurons, non-modulated neurons; F mean, the mean frequency; F burst, the burst frequency; F inter, the interburst frequency; Mod depth, the depth of modulation; n, the number of neurons. The values (mean ± s.e.m.) are given for all F-neurons, E-neurons and N/M-neurons (All), as well as for those located in each of three zones of the grey matter (Fig. 2). Indication of significance level: *P < 0.05; **P < 0.01; ***P < 0.001.

**Table 2:** Effects of spinalization on the efficacy of tilt-related sensory inputs from the ipsilateral and contralateral limbs to F- and E-neurons.

	**All**		**Zone 1**		**Zone 2**		**Zone 3**	
	**Control**	**Spinal**	**Control**	**Spinal**	**Control**	**Spinal**	**Control**	**Spinal**
Ipsi-tilts								
F-neurons	n = 175	n = 102	n = 42	n = 28	n = 62	n = 41	n = 71	n = 33
F mean	10.8 ± 0.6	8.0 ± 0.7**	11.5 ± 0.9	8.4 ± 1.2*	9.8 ± 1.1	8.5 ± 1.2	11.3 ± 1.1	6.8 ± 1.0**
F burst	16.2 ± 0.9	11.7 ± 1.0***	20.0 ± 1.7	14.0 ± 1.6**	15.0 ± 1.7	12.8 ± 1.9	15.1 ± 1.4	8.3 ± 1.1***
F interburst	5.5 ± 0.5	4.2 ± 0.6	3.1 ± 0.8	2.9 ± 1.0	4.6 ± 0.7	4.2 ± 1.0	7.6 ± 0.8	5.4 ± 0.9
Mod depth	10.8 ± 0.8	7.5 ± 0.9**	16.9 ± 1.9	11.1 ± 1.4*	10.4 ± 1.4	8.6 ± 1.7	7.4 ± 0.9	3.0 ± 0.6***
E-neurons	n = 132	n = 109	n = 32	n = 25	n = 45	n = 67	n = 55	n = 17
F mean	8.7 ± 0.6	6.6 ± 0.5**	9.5 ± 1.3	7.5 ± 0.7	8.5 ± 1.0	6.3 ± 0.7	8.4 ± 0.8	6.5 ± 1.5
F burst	12.4 ± 0.8	10.4 ± 0.8	14.7 ± 1.8	12.3 ± 1.2	12.6 ± 1.6	9.9 ± 1.1	10.9 ± 1.0	9.3 ± 1.7
F interburst	4.9 ± 0.5	2.8 ± 0.4***	4.2 ± 1.1	2.7 ± 0.6	4.3 ± 0.8	2.6 ± 0.5	5.8 ± 0.7	3.7 ± 1.6
Mod depth	7.4 ± 0.7	7.6 ± 0.7	10.5 ± 1.5	9.7 ± 1.3	8.2 ± 1.5	7.3 ± 1.0	5.1 ± 0.8	5.6 ± 1.2
Contra-tilts								
F-neurons	n = 175	n = 102	n = 42	n = 28	n = 62	n = 41	n = 71	n = 33
F mean	7.9 ± 0.6	5.3 ± 0.6**	7.6 ± 1.1	5.1 ± 1.1	7.2 ± 1.0	5.6 ± 1.1	8.7 ± 0.9	5.1 ± 0.9**
F burst	9.4 ± 0.7	5.8 ± 0.6***	8.7 ± 1.2	5.6 ± 1.1	8.6 ± 1.2	6.1.0 ± 1.1	10.6 ± 1.0	5.9 ± 0.9***
F interburst	6.4 ± 0.5	4.7 ± 0.6*	6.5 ± 1.1	4.5 ± 1.1	5.8 ± 0.9	5.1 ± 1.1	6.8 ± 0.8	4.4 ± 0.9*
Mod depth	3.1 ± 0.3	1.2 ± 0.2***	2.2 ± 0.5	1.0 ± 0.3	2.8 ± 0.7	1.0 ± 0.2**	3.8 ± 0.4	1.5 ± 0.3***
E-neurons	n = 132	n = 109	n = 32	n = 25	n = 45	n = 67	n = 55	n = 17
F mean	9.0 ± 0.6	4.6 ± 0.5***	9.2 ± 1.3	4.3 ± 0.7**	8.5 ± 1.2	4.5 ± 0.6**	9.2 ± 0.9	5.3 ± 1.5*
F burst	11.8 ± 1.0	5.1 ± 0.5***	12.4 ± 2.1	4.9 ± 0.8**	11.2 ± 2.0	4.9 ± 0.6**	11.9 ± 1.2	5.9 ± 1.5**
F interburst	6.1 ± 0.5	4.1 ± 0.5**	6.0 ± 1.0	3.6 ± 0.7	5.8 ± 0.9	4.0 ± 0.6	6.5 ± 0.7	4.7 ± 1.4
Mod depth	5.7 ± 0.8	1.1 ± 0.1***	6.4 ± 1.9	1.3 ± 0.5*	5.5 ± 1.9	0.9 ± 0.1*	5.4 ± 0.7	1.2 ± 0.3***

Designations: Ipsi-tilts, tilts of the ipsilateral platform only; Contra-tilts, tilts of the contralateral platform only. Other abbreviations as in Table 1.
